# Automated molecular detection of the vancomycin resistance genes vanA and vanB using the geneLEAD VIII platform

**DOI:** 10.1099/acmi.0.001044.v4

**Published:** 2025-08-07

**Authors:** Keiichiro Mori, Yasufumi Matsumura, Yusuke Tsuda, Koh Shinohara, Yasuhiro Tsuchido, Masaki Yamamoto, Miki Nagao

**Affiliations:** 1 Department of Clinical Laboratory, Kyoto University Hospital, Kyoto, Japan; 2 Department of Infection Control and Prevention, Kyoto University Hospital, Kyoto, Japan; 3 Department of Clinical Laboratory Medicine, Kyoto University Graduate School of Medicine, Kyoto, Japan

**Keywords:** automation, enterococci, real-time PCR, *vanA*, *vanB*, vancomycin-resistant enterococci (VREs)

## Abstract

Rapid and accurate detection of vancomycin-resistant enterococci (VREs) can aid in the early application of appropriate antimicrobial treatment and the implementation of infection prevention measures. The VIASURE real-time PCR assay (Certest Biotec) is a multiplex nucleic acid-based *in vitro* diagnostic test intended for the detection of *vanA* and *vanB*, and geneLEAD VIII (Precision System Science) is a customizable, fully automated molecular detection platform. We evaluated the performance of the VIASURE assay on the geneLEAD VIII platform against 200 clinical enterococcal isolates consisting of 151 VREs and 49 vancomycin-susceptible enterococci collected in Japan, primarily in the Kinki region, and compared it to that of the in-house reference multiplex PCR assay. The performance of the VIASURE assay for the detection of *vanA* and *vanB* was comparable to that of the reference multiplex PCR assay, with both a sensitivity and specificity of 100%. Compared with the reference PCR assay, the VIASURE assay reduced the turn-around time by ~3 h 40 min (5 h 34 min vs. 1 h 54 min) and the hands-on time by 46 min per four samples. Fully automated molecular detection of *vanA* and *vanB* using the VIASURE assay and geneLEAD VIII for bacterial isolates can enable fast and reliable testing while reducing labour and is a promising tool for clinical laboratories and nosocomial infection control.

## Data Summary

All data used in this study are available within the manuscript and supplemental materials.

## Introduction

Enterococci are commensal organisms that live in the human gastrointestinal tract, and they rarely cause infections in healthy individuals. However, some enterococci species*,* especially *Enterococcus faecalis* and *Enterococcus faecium*, which are one of the most commonly detected nosocomial pathogens, can cause severe hospital-associated infections in immunocompromised patients, such as those with cancer or serious underlying diseases [1,4]. Vancomycin-resistant enterococci (VREs) are considered a significant global threat associated with antimicrobial resistance [5], and the Centers for Disease Control and Prevention estimated that VREs caused 54,500 hospitalized infections and 5,400 deaths in the USA in 2017 [6]. Vancomycin resistance is also independently associated with increased mortality among patients with enterococcal bloodstream infections [7,9]. Rapid diagnostic testing and subsequent infection control for VREs decrease the time needed to initiate effective and appropriate antimicrobial therapy and reduce hospital costs [10,13].

The most prevalent vancomycin resistance mechanism of enterococci is the acquisition of the *vanA* or *vanB* genes [14,16]. The Clinical and Laboratory Standards Institute (CLSI) defines VREs as enterococci with a vancomycin MIC of ≥32 µg ml^−1^. Traditional culture and antimicrobial susceptibility testing-based methods often take several days to complete and may miss enterococci with vancomycin resistance genes [17,20] because of the presence of isolates with vancomycin MICs of <32 µg ml^−1^ [21]. Therefore, PCR-based detection assays targeting *vanA* and *vanB* have been developed and introduced into clinical laboratories [22,26]. Some of them run on a fully automated platform, providing results within an hour and allowing direct analysis of clinical specimens [24]. Direct detection of VREs offers a greater reduction in testing time by eliminating the need for bacterial culture. However, routine implementation of direct detection is limited by its high cost. Since culture remains essential for antimicrobial susceptibility testing and performing additional analyses such as strain typing, culture and molecular testing approaches cannot be entirely replaced. For these reasons, automation of molecular testing of VRE isolates remains an important strategy for enabling timely infection control interventions while minimizing the laboratory workload.

The VIASURE *Vancomycin resistance* Real Time PCR Detection Kit (Certest Biotec, Zaragoza, Spain), designed to detect *vanA* and *vanB*, has been commercially available in Spain and several European countries since 2018. geneLEAD VIII (Precision System Science, Chiba, Japan) is a customizable, fully automated molecular detection platform that can perform DNA extraction, amplification, real-time PCR and result interpretation with a maximum of eight samples per batch. The aim of the present study was to evaluate the performance of automated VIASURE assay testing on the geneLEAD VIII platform using clinical VRE isolates collected in Japan.

## Methods

### Bacterial isolates

A total of 200 clinical isolates of *Enterococcus* spp. (*E. faecium*, *Enterococcus gallinarum*, *E. faecalis*, *Enterococcus casseliflavus*, *Enterococcus avium*, *Enterococcus raffinosus*,* Enterococcus thailandicus* and *Enterococcus pallens*; Table 1), comprising 151 VRE isolates and 49 vancomycin-sensitive enterococci (VSE) isolates, were included. Among these, 148 VRE and 18 VSE isolates were collected from 39 facilities (hospitals, long-term care facilities and clinical laboratories) in Kyoto and 11 other prefectures of Japan during surveillance programmes performed since 2005 [27,28]. Three VRE and 31 VSE isolates were collected at Kyoto University Hospital, Kyoto, Japan, between 2021 and 2023. The VSE isolates collected at Kyoto University Hospital were randomly selected from 264 strains obtained from blood culture specimens. This study used only bacterial strains that had been anonymized and assigned unique research identification numbers with no linkage to patient-identifying information. VRE isolates were defined as isolates with a vancomycin MIC of ≥32 µg ml^−1^ and the presence of *vanA* and/or *vanB* according to the reference PCR assay. VSE isolates were defined as isolates with a vancomycin MIC of ≤16 µg ml^−1^. Antimicrobial susceptibility was evaluated by broth microdilution at each laboratory following the CLSI guideline M100-Ed35 [29]. Species identification was performed by MALDI-TOF MS using the MBT Compass software (version 4.1; Bruker Daltonics, Bremen, Germany). The isolates stored at −80 °C were cultured on TSA II 5% sheep blood agar M (Becton Dickinson, Tokyo, Japan). All plates were incubated aerobically at 35 °C for 48 h, and bacterial suspensions of 1.0 McFarland were prepared using 1 ml of saline solution (Eiken Chemical, Tokyo, Japan) for molecular testing. The same bacterial suspension was aliquoted and used for both the VIASURE assay and the reference PCR assay.

**Table 1.** Enterococcal isolates used in this study and the results of the reference PCR assay

Species	Total (*n*=200)	VRE* (*n*=151)		VSE* (*n*=49)
		*vanA^＋^*, *vanB^−^* (*n*=76)	*vanA^−^*, *vanB^＋^* (*n*=75)	*vanA^−^*, *vanB^−^*
***E. faecium***	71 (35.5)	42 (55.3)	20 (26.7)	9 (18.4)
***E. gallinarum***	58† (29.0)	31 (40.8)	20 (26.7)	7 (14.3)
***E. faecalis***	44 (22.0)	0 (0)	30 (40.0)	14 (28.6)
***E. casseliflavus***	12‡ (6.0)	0 (0)	5 (6.7)	7 (14.3)
***E. avium***	8 (4.0)	3 (3.9)	0 (0)	5 (10.2)
***E. raffinosus***	5 (2.5)	0 (0)	0 (0)	5 (10.2)
***E. thailandicus***	1 (0.5)	0 (0)	0 (0)	1 (2.0)
***E. pallens***	1 (0.5)	0 (0)	0 (0)	1 (2.0)

*Superscript plus symbols indicate the presence of the gene, and superscript minus symbols indicate its absence.

†*vanC1* was detected in 57 isolates (98.3%).

‡*vanC2/3* was detected in seven isolates (58.3%).

### VIASURE assay

**Fig. 1.**
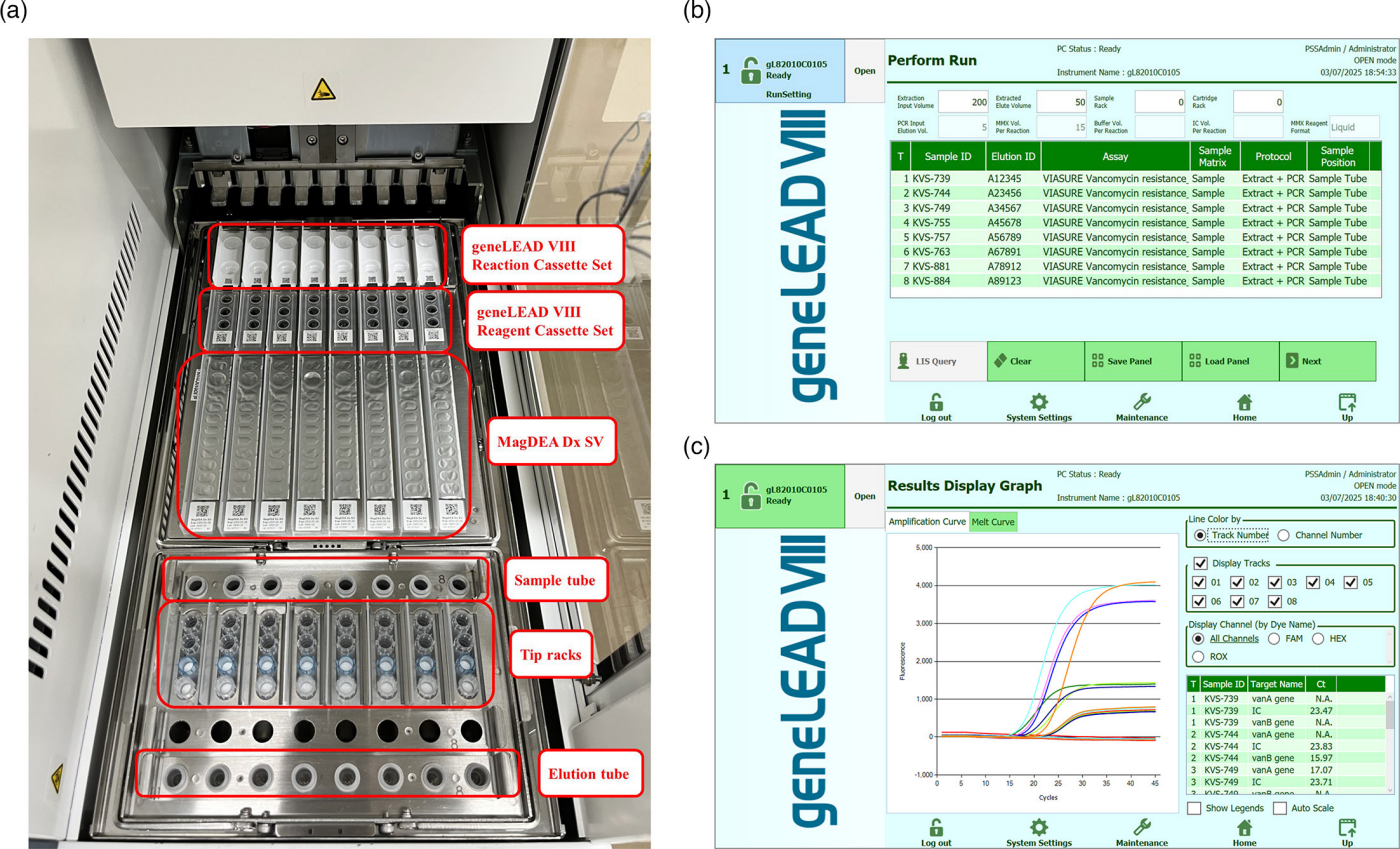
Preparation of the instrument and results of the VIASURE assay using the geneLEAD VIII platform. (**a**) Arrangement of tubes, tip racks and reagents on the geneLEAD VIII deck. (**b**) The geneLEAD VIII screen showing the assay setup. Sample and elution tube identifiers were entered using a barcode reader. After the assay name was selected, geneLEAD VIII guided the deck setup process shown in panel (a). (**c**) The geneLEAD VIII screen showing the results. Amplification curves and *C_T_* values of *vanA*, *vanB* and internal control targets are displayed. Assay interpretations are also displayed (not shown).

DNA was extracted from 200 µl of bacterial suspension using MagDEA Dx SV (Precision System Science) and eluted in 50 µl. The real-time PCRs were performed with a VIASURE *Vancomycin resistance* Real Time PCR Detection Kit (Certest Biotec), which targets *vanA*,* vanB* and internal controls, according to the manufacturer’s protocol. Briefly, 390 µl of rehydration buffer was added to the reaction mixture tube to rehydrate the lyophilized reagent. Seventeen microlitres of the reaction mixture were dispensed into a geneLEAD VIII PCR Reagent Cassette (Precision System Science) per isolate, of which 15 µl of the reaction mixture was used for real-time PCR. The cycling protocol consisted of 1 cycle of 2 min at 95 °C followed by 45 cycles of denaturation for 10 s at 95 °C, annealing and extension for 50 s at 60 °C. Cycle threshold (*C_T_*) values of <40 for *vanA* and/or *vanB* were considered positive for the vancomycin resistance genes. *C_T_* values of ≥40 for *vanA* and *vanB* were considered negative if the *C_T_* values for the internal control were in the valid range of <40. The negative and positive controls of the kit were used as control samples for each run. The negative controls were loaded as 200 µl samples on the instrument. The positive controls were dissolved in 100 µl of nuclease-free water, 20 µl of which was diluted tenfold with nuclease-free water and loaded onto the instrument as samples. Preparation for automated testing was conducted following geneLEAD VIII software, which guides the placement of 1.5 ml tubes containing bacterial suspensions or controls, elution tubes, tip racks, MagDEA Dx SV extraction reagent cartridges, a geneLEAD VIII Reaction Cassette (PCR chamber; Precision System Science) and a geneLEAD VIII Reagent Cassette containing the reaction mix at appropriate positions on the deck (Fig. 1). The entire testing process, including interpretations of the test results, was automatically performed. The run time of one batch (up to eight samples) was ~100 min.

### Reference PCR assay

DNA was obtained from 200 µl bacterial suspensions using a QIAamp DNA Mini Kit (Qiagen, Hilden, Germany). The extracted DNA was eluted with 100 µl of elution buffer. In-house multiplex PCR was performed according to a previous report [30] using *TaKaRa Ex Taq* (Takara Bio, Shiga, Japan). This assay detects seven different genes: *vanA*, *vanB*, *vanC1*, *vanC2/C3*, * ddl_E. faecalis_*, *ddl_E. faecium_* and *rrs* (16S rRNA gene of enterococci). We used *vanA*-positive *E. faecium* (FN1), *vanB*-positive * E. faecalis* (WBH13116), *vanC1*-positive *E. gallinarum* (H51) and *vanC2*-positive *E. casseliflavus* (C41) as positive controls and nuclease-free water as a negative control.

### Test time measurement

The time required to perform each test step for the testing of four samples was measured in triplicate for the VIASURE assays and the reference PCR assay. Trials were performed by the same skilled operator on different days. The turn-around time was defined as the time from the preparation of the bacterial suspensions to the availability of the test results.

### Statistical analysis

For *vanA* and *vanB*, the agreement between the VIASURE and reference PCR assays was assessed with positive percentage agreement (PPA), negative percentage agreement (NPA) and overall percentage agreement (OPA). PPA and NPA (with two-sided 95% CIs) were calculated for each vancomycin-resistant genotype and compared via the McNemar test. Cohen’s kappa coefficient was calculated to assess the overall agreement between the VIASURE and reference PCR assays. A *P* value less than 0.05 was considered statistically significant for all analyses. All the statistical analyses were performed using Stata, version 13.1 (StataCorp, College Station, TX).

## Results

Eight species of enterococci were represented in the 200 study isolates: *E. faecium* (*n*=71, 35.5%), *E. gallinarum* (*n*=58, 29.0%), *E. faecalis* (*n*=44, 22.0%), *E. casseliflavus* (*n*=12, 6.0%) and other species (*n*=15, 7.5%) (Table 1). Among the 151 VRE isolates, the reference PCR assay detected *vanA* in 76 isolates and *vanB* in 75 isolates. The numbers and percentages of VRE isolates positive for *vanA*/*vanB* in each species were as follows: *E. faecium* (*n*=62, *vanA*/*vanB*: 67.7/32.3%), *E. gallinarum* (*n*=51, 60.8/39.2%), *E. faecalis* (*n*=30, 0/100%), *E. casseliflavus* (*n*=5, 0/100%) and *E. avium* (*n*=3, 100/0%). *vanC1* and *vanC2/3* were detected in only *E. gallinarum* and *E. casseliflavus*, respectively. No vancomycin resistance genes (*vanA*, *vanB*, *va*nC1 or *vanC2/3*) were detected in the 41 VSE isolates. The results of the VIASURE and reference PCR assay were completely concordant. For both *vanA* and *vanB*, PPAs, NPAs, OPAs and Cohen’s kappa values were 100% (95% CI, 95.2–100%), 100% (95% CI, 97.0–100%), 100% and 1, respectively. The PPA and NPA values did not differ significantly. All test results for each isolate, including the *C_T_* values obtained from the VIASURE assay, are provided in Dataset S1, available in the online Supplementary Material 1.

**Fig. 2.**
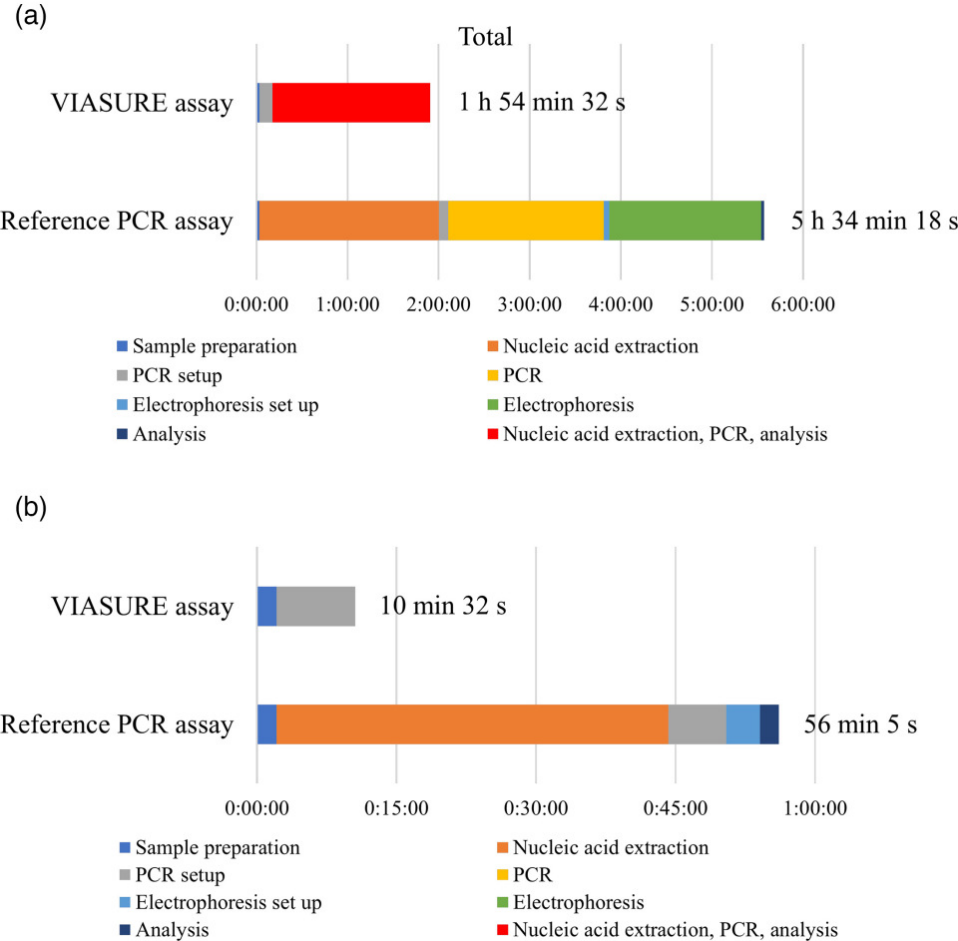
Comparison of average test times for each test step between the VIASURE assay and the reference PCR assay. Panel (a) shows the turn-around time, and panel (b) shows the hands-on time.

The average turn-around time of the reference PCR assay was ~5 h 34 min for four samples, and the hands-on time was 56 min (Fig. 2 and Dataset S2). In contrast, the turn-around time of the VIASURE assay was ~1 h 54 min. The hands-on time was 10 min, and the remaining time was the run time of geneLEAD VIII. Compared with the reference PCR assay, the VIASURE assay reduced the turn-around time by ~3 h 40 min and the hands-on time by 46 min.

## Discussion

The international prevalence of vancomycin-resistant *E. faecium* has been increasing; the European Antimicrobial Resistance Surveillance Network and the Central Asian and European Surveillance of Antimicrobial Resistance surveillance programmes show that vancomycin resistance rates in *E. faecium* increased from 15.0% in 2017 to 17.2% in 2021 [31]. *E. faecium* is also the most frequently reported VRE strain in Japan [32]. The most dominant vancomycin resistance gene type (i.e. *vanA* or *vanB*) varies from country to country [33,38], and in some countries, the predominant type has changed over time [16,39,41]. Several outbreaks caused by VRE strains with *vanA* have been reported in Japan [28,42,44], but the number of VRE strains with *vanA* and *vanB* in this study was similar. This can be explained by the fact that most of the study isolates were derived from surveillance programmes, mainly in the Kyoto region, reflecting the local epidemiological patterns [27,28]. None of the VRE isolates studied carried both *vanA* and *vanB*, although such strains have been reported outside Japan (e.g. Denmark, Greece and Vietnam) [39,45, 46].

The 100% concordance was observed between the VIASURE and reference PCR assays. This may have been influenced by the use of bacterial colonies rather than clinical specimens, the use of the same bacterial suspension for both assays and the fact that many of the VRE isolates originated from regional outbreaks, potentially leading to uniform genetic profiles, in addition to the implementation of standardized and quality-controlled laboratory procedures. 

Comparative characteristics of molecular assays using fully automated molecular detection platforms are shown in Table S1. These assays target both *vanA* and *vanB* [22,26]. In addition to geneLEAD VIII, fully automated molecular detection platforms that can automate custom in-house assays include the cobas 5800/6800/8800 systems (Roche, Basel, Switzerland) and the BD MAX system (Becton Dickinson). These customizable platforms can preserve extracted nucleic acids, which can be used for further genetic investigations. Assays other than the VIASURE assay listed in Table S1 have been evaluated for their performance in the direct detection of *vanA* and *vanB* in clinical samples (rectal swab or positive blood culture). The processing mode (batch or random access), testing capacity (1–96 samples) and turn-around time (1–6 h) varied among the assays. The maximum testing capacity of geneLEAD VIII (eight samples) is the lowest among the batch processing-type instruments, which is a potential limitation, although the capacity may be sufficient for most hospital laboratory settings. The running cost of the VIASURE assay is approximately €34/sample, including the costs of the controls and consumables. This is cheaper than other proprietary systems [e.g. Xpert *vanA/vanB* (Cepheid, Sunnyvale, CA, USA), €51/sample, and BioFire FilmArray Blood Culture Identification 2 Panel (bioMérieux, Marcy-l'Étoile, France), €121/sample]. These estimations were based on Japanese market prices, converted to euros at an exchange rate of 1€=160 yen. In a recent survey, Leber *et al.* reported that more than 80% of microbiology laboratories are understaffed and that these laboratories are difficult to fill for a variety of reasons [47]. However, laboratory automation can not only reduce turn-around time and increase productivity but also improve quality and reduce labour and costs [48,49]. The results of this study show that the automated VIASURE assay reduced the workload while shortening the turn-around time and enabling high-quality molecular testing.

Our study has several limitations. First, the isolates were collected only in Japan and were biassed toward regional strains. Second, the study was conducted at a single institution, limiting the generalizability of the results. Third, the performance of the VIASURE–geneLEAD combination assay for the direct detection of VRE from clinical samples was not evaluated.

In conclusion, the results of the present study suggest that the VIASURE assay using geneLEAD VIII has excellent sensitivity and specificity for the detection of VRE from bacterial suspensions. The use of this assay with geneLEAD VIII, which allows automated extraction of nucleic acids followed by real-time PCR, could have important implications for clinical laboratories and infection control due to its shorter turn-around and hands-on time compared to that of manual PCR testing.

## Supplementary material

10.1099/acmi.0.001044.v4 Table S1.

10.1099/acmi.0.001044.v4 Supplementary Material 1.

## Abbreviations

CI: confidence interval
CLSI: Clinical and Laboratory Standards Institute
*C_T_*: cycle threshold
MIC: minimum inhibitory concentration
NPA: negative percentage agreement
OPA: overall percentage agreement
PPA: positive percentage agreement
VRE: vancomycin-resistant enterococci
VSE: vancomycin-sensitive enterococci
