# Supplementary material for: Automated molecular detection of the vancomycin resistance genes vanA and vanB using the geneLEAD VIII platform
Source: Access Microbiol. 2025 Aug 7;7(8):001044.v4. doi: 10.1099/acmi.0.001044.v4 (PMC12451298; doi:10.1099/acmi.0.001044.v4)
Supplement: Table S1. [file acmi-7-01044-s001.pdf]

Automated molecular detection of the vancomycin resistance genes *vanA* and *vanB* using the geneLEAD VIII platform

Supplementary Material

Table S1. Comparative characteristics of the six PCR assays with fully automated molecular detection platforms for the detection of *vanA* and *vanB*

**Characteristic**	**Assay name**					
	**VIASURE *Vancomycin resistance* Real Time PCR Detection Kit**	***vanA/B* UTC**	**BD Max VRE PCR**	**cobas ePlex blood culture identification gram-positive panel**	**Xpert *vanA/vanB***	**BioFire FilmArray Blood Culture Identification 2 Panel**
**Assay manufacturer**	Certest Biotec	In house	In house	Roche	Cepheid	bioMérieux
**Specimens**	Bacterial suspension	Rectal swab[a] (eSwab, Copan), VRE enrichment in broth	Rectal swab[a] (eSwab, Copan)	Positive blood culture	Bacterial suspension, rectal swab[a] (eSwab, Copan), enriched inoculated broth	Positive blood culture

**Automated molecular detection platform**	geneLEAD VIII (PSS)	cobas 5800/6800/8800 systems (Roche)	BD MAX system (BD)	cobas ePlex system (Roche)	GeneXpert system (Cepheid)	BioFire FilmArray instrument (bioMérieux)
**Processing mode**	Batch	Batch	Batch	Random access	Random access	Random access
**Test capacity**[b]	8 samples per run[c]	24 samples per run for cobas 5800 system, 96 samples per run for cobas 6800/8800 systems	24 samples per run	Single test (Up to 24 test bays)	Single test (Up to 80 modules)	Single test (Up to 12 modules)
**Regulatory status**	*In vitro* diagnostic test	Laboratory developed test	Laboratory developed test	*In vitro* diagnostic test	*In vitro* diagnostic test	*In vitro* diagnostic test

**Samples to be prepared before analyzing**	Isolate suspended in 1 ml saline solution	Rectal swab diluted 1:6 with cobas PCR medium, 2 ml of VRE enriched broth containing 100 µl of Amies medium	Not required	Not required	Bacterial solution diluted in water added to 75 µl elution buffer, 100 µl rectal swab/enriched inoculated broth added to elution buffer	200 µl positive blood culture with dilution buffer
**Sample volume for testing**	200 µl	400 µl	150 µl	50 µl	100 µl	200 µl
**Turn-around time per run**	1 h 55 min	6 h[d]	NR	90 min	Within 1 h	Within 2 h
**Hands-on time**	15 min	NR	Less than 15 min	NR	NR	NR

			per 8 samples			
**Reference**	This study	Giersch et al. (1)	Dalpke et al. (2)	Tansarli et al. (3)	Zhou et al. (4)	El Sherif et al. (5)

PSS, Precision System Science; BD, Becton Dickinson; NR, not reported.

[a] eSwab combines a flocked swab with 1 mL of Liquid Amies in a plastic, screw cap tube.

[b] Data were collected using the platform instruction manuals.

[c] When measuring controls, up to 6 samples can be measured per run.

[d] Turn-around time if the samples were rectal swabs.

References

1. Giersch K, Tanida K, Both A, Nörz D, Heim D, Rohde H, Aepfelbacher M, Lütgehetmann M. Adaptation and validation of a quantitative *vanA/vanB* DNA screening assay on a high-throughput PCR system. Sci Rep. 2024 Feb 12;14(1):3523.
2. Dalpke AH, Hofko M, Zimmermann S. Development of a Real-Time PCR Protocol Requiring Minimal Handling for Detection of Vancomycin-Resistant Enterococci with the Fully Automated BD Max System. J Clin Microbiol. 2016 Sep;54(9):2321-9.
3. Tansarli GS, Chapin KC. A Closer Look at the Laboratory Impact of Utilizing ePlex Blood Culture Identification Panels: a Workflow Analysis Using Rapid Molecular Detection for Positive Blood Cultures. Microbiol Spectr. 2022 Oct 26;10(5):e0179622.
4. Zhou X, Arends JP, Kampinga GA, Ahmad HM, Dijkhuizen B, van Barneveld P, Rossen JW, Friedrich AW. Evaluation of the Xpert *vanA/vanB* assay using enriched inoculated broths for direct detection of *vanB* vancomycin-resistant Enterococci. J Clin Microbiol. 2014 Dec;52(12):4293-7.
5. El Sherif HM, Elsayed M, El-Ansary MR, Aboshanab KM, El Borhamy MI, Elsayed KM. BioFire FilmArray BCID2 versus VITEK-2 System in Determining Microbial Etiology and Antibiotic-Resistant Genes of Pathogens Recovered from Central Line-

Associated Bloodstream Infections. Biology (Basel). 2022 Oct 26;11(11):1573.
